# Structural Insights into the Heme Pocket and Oligomeric State of Non-Symbiotic Hemoglobins from *Arabidopsis thaliana*

**DOI:** 10.3390/biom10121615

**Published:** 2020-11-29

**Authors:** Alessandra Astegno, Carolina Conter, Mariarita Bertoldi, Paola Dominici

**Affiliations:** 1 Department of Biotechnology, University of Verona, Strada Le Grazie 15, 37134 Verona, Italy; alessandra.astegno@univr.it (A.A.); carolina.conter@univr.it (C.C.); 2 Department of Neuroscience, Biomedicine and Movement Sciences, Section of Biological Chemistry, University of Verona, Strada Le Grazie, 8, 37134 Verona, Italy; mita.bertoldi@univr.it

**Keywords:** non-symbiotic hemoglobins, *Arabidopsis thaliana*, circular dichroism, hexacoordination, quaternary structure

## Abstract

Non-symbiotic hemoglobins AHb1 and AHb2 from *Arabidopsis thaliana* are hexacoordinate heme-proteins that likely have different biological roles, in view of diverse tissue localization, expression pattern, and ligand binding properties. Herein, we expand upon previous biophysical studies on these isoforms, focusing on their oligomeric states and circular dichroism (CD) characteristics. We found that AHb1 exists in solution in a concentration-dependent monomer-dimer equilibrium, while AHb2 is present only as a monomer. The quaternary structure of AHb1 affects its degree of hexacoordination with the formation of the dimer that enhances pentacoordination. Accordingly, the mutant of a conserved residue within the dimeric interface, AHb1-T45A, which is mostly monomeric in solution, has an equilibrium that is shifted toward a hexacoordinate form compared to the wild-type protein. CD studies further support differences in the globin’s structure and heme moiety. The Soret CD spectra for AHb2 are opposite in sense to those for AHb1, reflecting different patterns of heme-protein side chain contacts in the two proteins. Moreover, the smaller contribution of the heme to the near-UV CD in AHb2 compared to AHb1 suggests a weaker heme-protein association in AHb2. Our data corroborate the structural diversity of AHb1 and AHb2 and confirm the leghemoglobin-like structural properties of AHb2.

## 1. Introduction

Hemoglobins (Hbs) represent a large family of globular proteins that exist in nearly all organisms, from bacteria to higher eukaryotes [1], and participate in a broad variety of biological functions. Most of these functions are tightly associated with the prosthetic heme iron reactivity towards small ligands, especially oxygen.

Plant genomes, besides the well-characterized symbiotic hemoglobins (e.g., leghemoglobins) contain multiple non-symbiotic hemoglobins (nsHbs) [2]. Symbiotic hemoglobins possess properties of oxygen transport hemoglobins as they are present in millimolar concentrations, have moderate affinity for oxygen (*K_d_* ≈ 50 nM), a quite rapid dissociation rate constant for oxygen (~5–15 s^−1^), and are pentacoordinate [3]. These properties facilitate their function in transporting oxygen in the root nodules of plants for symbiotic nitrogen fixation. In comparison, a remarkable feature of the nsHbs is the heme hexacoordination resulting from the presence of the proximal F8 and the distal E7 histidine residues that occupy the fifth and sixth coordination position, respectively, of the heme iron. In nsHbs, the hexacoordination is reversible, thus allowing for the rapid binding of ligands such as O_2_, NO, and CO with high affinity. For this reason, the structures and chemistries of nsHbs have been the subject of much attention during past years due to possible functions in sensing and detoxifying NO or in responding to other environmental stresses.

The nsHbs are organized into three different classes: class 1 (nsHb1), class 2 (nsHb2), and class 3, known as truncated hemoglobins (trHbs) [4]. All three classes of nsHbs are present in *Arabidopsis thaliana*, namely AHb1, AHb2 and AHb3, and each possesses specific ligand binding and structural properties that are indicative of their different biological roles [4,5,6]. AHb1 and AHb2 differ markedly in their expression patterns and tissue localization; AHb1 is expressed in rosette leaves and roots in response to hypoxic conditions [6] and upon exposure to either sucrose or nitrate [7], while AHb2 is found in rosette leaves and is overexpressed at low temperatures [6]. Both proteins show a characteristic 3/3 α-helical fold. However, AHb1 has the typical sequence and oxygen-binding properties of nsHb proteins, while the sequence and rate constants of oxygen-binding for AHb2 are more similar to those of leghemoglobins and symbiotic hemoglobins [6,8,9]. Indeed, AHb1 possesses a high affinity for oxygen (*K_d_* ≈ 2–10 nM), moderate rates of oxygen binding (~74 µM^−1^ s^−1^), and a small dissociation rate constant for oxygen (~0.12 s^−1^) [5,6,10]. It is believed to have a role in NO detoxification by working as a NO dioxygenase, and therefore decreasing the NO levels under hypoxic conditions [11]. In comparison, AHb2 possesses lower oxygen affinity (*K_d_* ≈ 100–200 nM), low rates of oxygen binding (~1 µM^−1^ s^−1^), relatively low dissociation rate constants for oxygen (~0.14 s^−1^) [5,6], and has been suggested to function in oxygen sensing, storage, and diffusion [12]. The similarity of the oxygen-binding properties of class 2 nsHbs, including AHb2, to the symbiotic group has supported the hypothesis that class 2 hemoglobins are the direct ancestor of leghemoglobins, which have acquired precise properties in oxygen homeostasis for symbiosis with nitrogen-fixing bacteria [4,6,13,14].

Another striking difference that distinguishes class 1 and class 2 nsHbs is the weaker hexacoordination (K_HisE7_ ≈ 2) of class 1 nsHbs compared to class 2 nsHbs (K_HisE7_ ≈ 100). As such, AHb2 is fully hexacoordinate, while AHb1 exists as a mixture of penta- and hexacoordinate heme and the two isoforms display distinct interactions between the residues in the distal cavity, heme, and ligands [15,16,17]. The different reactivity with ligands has been proposed to be associated with the presence of a diverse arrangement of hydrophobic cavities in AHb1 and AHb2, arguing in favor of their distinct functions [10,16,18]. In particular, the presence of a relevant fraction of unliganded pentacoordinate AHb1 promotes the entrance of ligands into the distal cavity via a distal histidine (HisE7) gating mechanism [17], as was already observed in carboxymyoglobin [19]. In AHb1, the distal cavity is linked to the solvent via a rather open channel, while in AHb2 migration of ligand from the distal heme cavity to the exterior is influenced by protein dynamics. Two CO docking sites were identified in AHb1 by spectroscopic studies [18], and only one can be occupied in AHb2. Based on these findings, a structural explanation for the proposed role as a NO scavenger of AHb1 has been proposed. Oxygen binding causes the formation of a channel from the distal cavity to the exterior, allowing the occupation by the NO molecule of a docking site close to the heme-bound O_2_, thus favoring the reaction of the two molecules to produce nitrate. The lack of comparable internal structures in AHb2 suggests that this isoform is not involved in NO detoxification processes.

Knowledge of class 3 plant truncated Hbs is still limited. Class 3 nsHbs are described as pentacoordinate in the deoxy state and the typical distal histidine is missing. AHb3 possesses low similarity to class 1 and 2 nsHbs and displays a 2-on-2 arrangement of α-helices with the presence of an α-helical extension at the N-terminus and a disordered C-terminal region, distinct from the 3/3-fold of the other globins [5,20]. It is expressed in shoot roots and is downregulated by hypoxia. Moreover, it was found to exhibit low affinity for O_2_ (*K*_m_ ≈ 1500 nM) [5].

The crystal structure of AHb1 was reported by Mukhi et al. in 2013 [21]. The structure shows a conserved 3/3 globin fold with eight α-helices, named A to H. Like other members of class 1 nsHbs, AHb1 is a homodimer with the two chains in the hexacoordination state. Dimerization is promoted by both electrostatic and hydrophobic interactions between amino acids of the BC and G helices of each chain. Of note, these residues are highly conserved among class 1 nsHbs, supporting the hypothesis that the dimeric state could have an impact on the chemical behavior and role of this class of proteins. On the other hand, the X-ray structure of AHb2 has not yet been determined, while the crystal structure of the truncated AHb3 has recently been solved [20,22].

It is important to note that although much information has been obtained on the ligand binding properties of nsHbs, relating them with some structural features, a global picture of how the tertiary and quaternary structures impact the properties of each isoform is still missing.

In an attempt to obtain additional insights into the structure–function and evolutionary relationships of AHb1 and AHb2 and to identify specific structural fingerprints of these isoforms, we analyzed the heme moiety of AHb1 and AHb2 by circular dichroism (CD) spectroscopy and explored the oligomeric state in solution of the two isoforms by size exclusion chromatography (SEC). Moreover, site-directed mutagenesis of residue Thr45, impairing a critical interaction in the dimeric interface of AHb1, was applied to determine its effect on the oligomeric state and hexacoordination equilibrium of the protein.

## 2. Materials and Methods

### 2.1. Chemicals

All chemicals were purchased from Sigma-Aldrich (Milano, Italy), unless otherwise stated.

### 2.2. Protein Production and Sample Preparation

AHb1, AHb2 and AHb1 E7L variants were purified as previously described [15,16,23]. AHb1 T45A mutant was made on the wild-type pET11a-AHb1 construct using the QuikChange II mutagenesis kit (Agilent Technologies, Santa Clara, CA, USA), according to the manufacturer’s recommendations. The conditions for expression and purification of the mutant were as described for the wild-type protein [15].

Ferrous-deoxy samples were prepared by adding sodium dithionite, while ferric proteins were obtained by adding potassium ferricyanide and elimination of excess ferricyanide using concentrating filters.

The apo-AHb1 was obtained by purification from *E. coli* without the addition of hemin. Spectroscopic measurement indicated that the percentage of apo-protein in the obtained sample was >70%.

### 2.3. Spectroscopic Measurements

Absorbance spectra were recorded at room temperature on a JASCO V560 Spectrophotometer (JASCO Corporation, Hachioji, Tokyo, Japan) in 20 mM Tris-HCl pH 8. For estimation of the fraction of pentacoordinate species at different concentrations of ferrous-deoxy wild-type AHb1 and T45A mutant, spectra of the proteins were recorded in 0.1 M phosphate buffer, pH 7.0, at protein concentration from 0.7 to 350 μM using 1-cm and 0.1-cm quartz cells.

CD spectra were measured at 25 °C on a JASCO J-1500 spectropolarimeter (JASCO Corporation, Hachioji, Tokyo, Japan) equipped with a thermostatically controlled sample compartment. CD spectra were recorded using a quartz cell of 1-cm path length (near-UV, Soret, and visible region), 50 nm/min scanning speed, 1-nm spectral bandwidth, and 1-nm data pitch, over the wavelength range from 240 to 650 nm. The CD spectra represent an average of three accumulations. All experiments were done by diluting a freshly prepared sample in 10 mM Tris-HCl pH 8. The concentrations of all protein samples were 30–40 μM in the near-UV and Soret region; while higher concentrations, i.e., 120 μM (AHb1) and 140 μM (AHb2), were used in the visible region. Solvent spectra were recorded and considered as a blank. A two-point and ten-point adjacent-averaging smoothing was performed for the near-UV and for the Soret and visible region CD spectra, respectively, with Origin Software.

Far-UV (250–190 nm) CD spectra were measured in 10 mM Tris-HCl pH 8 at a protein concentration of 0.2 mg/mL at 25 °C using a 0.1 cm quartz cell. Thermal unfolding profiles were obtained by following the CD signal at 222 of 0.2 mg/mL proteins in a temperature range between 20 and 100 °C (scan rate 1.5 °C/min) [24,25]. Mean values ± the standard error of the mean (SEM) of the melting temperature were obtained from triplicate experiments.

### 2.4. Size Exclusion Chromatography

The quaternary structure of AHb variants was analyzed by size exclusion chromatography (SEC) using a Superdex 75 Increase 10/300 GL column (GE Healthcare, Europe GmbH, Milano, Italy). The injection volume was 100 μL and the flow rate was 0.1 mL/min. The mobile phase was 150 mM Tris-acetate buffer, pH 7.5. A calibration curve was generated using the following standard proteins: bovine serum albumin (66 kDa), ovoalbumin (43 kDa), carbonic anhydrase (29 kDa), myoglobin (17.6 kDa) and cytochrome c (12.4 kDa). Blue dextran and acetone were also used in column calibration [26,27].

### 2.5. Evaluation of Dimer Dissociation Constant (K_d_) Values

The equilibrium constant for dimer dissociation was determined according to the method of Manning [28], adapted to the dimer-monomer equilibrium. This is a treatment that mathematically relates the protein concentration (in terms of the theoretical maximum concentration of dimer) to the expected amounts of dimer and monomer for an associating–dissociating equilibrium.

The percentage of dimer (%*D*) of each sample was calculated from the elution curves following the method of Manning et al. [28]. Assuming that the elution volume (*V_e_*) varies as a function of the molecular weight (*MW*) it follows that:

$$\frac{\log2}{V_{m}-V_{d}}=\;\frac{\log\frac{MW}{A}}{V_{m}-V_{e}}$$

where *A* is the *MW* of the monomer, and *V_d_* and *V_m_* are the elution volumes of the dimeric and monomeric species, respectively. At a given enzyme concentration the effective *MW* is given by:

$$MW=A\left(1+\frac{\%D}{100}\right)$$

where %*D* is the percentage of the dimer present. Combining the two equations gives:

$$\%D=100\left(2^{\left(V_{m}-V_{e})/(V_{m}-V_{d}\right)}-1\right)$$

The monomer-dimer equilibrium dissociation constant (*K_d_*) was calculated as follows.

$\left[D_{TOT}\right]$ denotes the maximal amount of AHb1 dimer, $[D_{TOT}]=\;\frac{\left[M\right]}{2}+\left[D\right],$ where [*D*] and [*M*] denote the concentrations of dimeric and monomeric species, respectively. The dimer dissociation constant for AHb1, $K_{d}=\;\frac{{\left[M\right]}^{2}}{\left[D\right]}$, can be estimated as follows [28]:

Given the following expressions:

$$\%D=100\;\frac{\left[D\right]}{\left[D_{TOT}\right]}\;\mathrm{and}\;\left[M\right]=2\left(\left[D_{TOT}\right]-\left[D\right]\right)$$

*K_d_* can be expressed as

$$K_{d}=\;\frac{2^{2\;}{\left(\left[D_{TOT}\right]-\left[D\right]\right)}^{2}}{\left[D\right]}=\left[D_{TOT}\right]\frac{0.04\;{\left(100-\%D\right)}^{2}}{\%D}$$

Hence, by applying the logarithm:

$$\log\left(K_{d}\right)=\log\left[D_{TOT}\right]-\log\;(\frac{\%D}{0.04{\left(100-\%D\right)}^{2}})$$

A plot of $\log\;(\frac{\%D}{0.04{\left(100-\%D\right)}^{2}})$ with respect to $\log\left[D_{TOT}\right]$ yields a straight line of slope 1.

When $\log\;(\frac{\%D}{0.04{\left(100-\%D\right)}^{2}})=0\;\to K_{d}=\left[D_{TOT}\right].$

The protein sample concentration loaded in SEC was diluted during separation. Therefore, $\left[D_{TOT}\right]$ must be divided by the dilution factor introduced during gel filtration. The justification for this correction can be found in references [28,29]. With a 100-μL sample load, the dilution factor during elution was measured by the peak width at half-height (mL) divided by the sample load volume (100 μL). Within experimental error, the peak widths at half height were constant over the range of AHbs concentrations, and the peak heights were also found to be related directly to the concentration of AHb injected. The dilution factor was found to be 8 ± 1 for ferrous-oxy AHb1 and ferric AHb1, 7 ± 1 for ferrous-oxy AHb1 T45A mutant, and 6 ± 1 for ferric AHb2. These values were constant over the entire AHbs concentrations range used. Each experiment was performed at least in triplicate using different batches of protein purified separately. Data were analyzed using Origin software and expressed as the mean ± SEM.

### 2.6. Native PAGE

Native protein electrophoresis (PAGE) and Ferguson plot analyses were performed to investigate the oligomeric state of native AHb2 [30,31]. The protein was electrophoresed in four parallel non-denaturating gels at 8, 9, 10, and 12% acrylamide/bis-acrylamide concentration (%T) and the relative mobility (Rf) was measured for each sample relative to the tracking dye. Retardation coefficients (Kr) were calculated from the slope of plot 100*log (100*Rf) against the %T. The Ferguson plot was constructed by plotting the log of the negative slope against the log of molecular mass to obtain a standard curve [30]. The following proteins were used as standards: α-Lactalbumin (14.2 kDa), carbonic anhydrase (29 kDa), chicken egg albumin (45 kDa), and bovine serum albumin (monomer 66 kDa and dimer 132 kDa).

## 3. Results

### 3.1. Oligomeric State of AHb1 and AHb2

The oligomeric state of recombinant AHb1 and AHb2 in solution was analyzed by SEC, since it is known that the quaternary structure affects the function of many hemoglobins.

**Figure 1:**
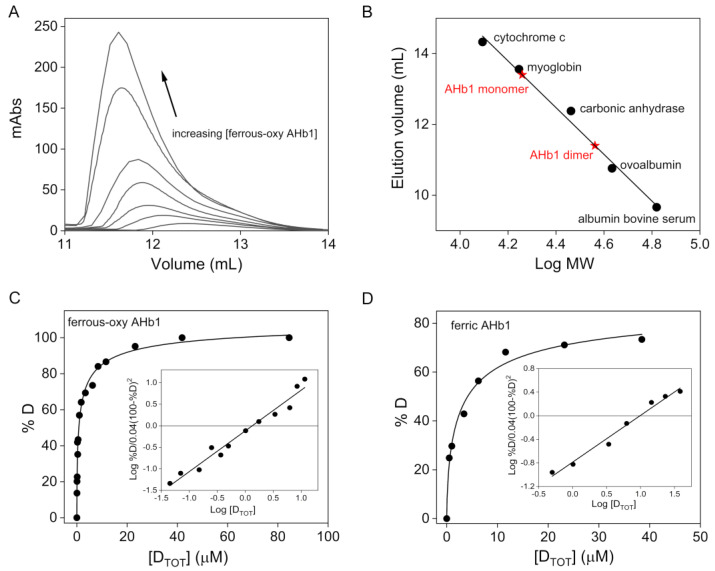
Oligomeric state of AHb1. (**A**) Representative elution profiles of ferrous-oxy AHb1 at selected protein concentrations (70 nM–85 μM range). The arrow indicates the direction of shift of the chromatographic peak with increasing protein concentration. (**B**) Calibration curve for standard proteins on a Superdex-75 Increase 10/300 GL column prepared by plotting the log MW value for each standard versus its corresponding elution volume value. The graph was used to determine the apparent MW of AHb1 monomer and dimer (red five-pointed stars). (**C**,**D**) Representative dimer-monomer dissociation curves of ferrous-oxy (**C**) and ferric (**D**) AHb1 in 150 mM Tris-acetate buffer, pH 7.5. [D_TOT_] represents the total AHb1 concentration (in dimer equivalents) and %D represents the percentage of protein that is actually dimer at various AHb1 concentrations. Inset of C and D shows the plot of log %D/0.04 (100 − %D)^2^ versus log [D_TOT_] of ferrous-oxy and ferric AHb1, respectively, according to Manning et al. [28]. Procedures used for evaluation of *K_d_* values are described in detail in Section 2.5.

At physiological pH (pH 7.5), ferrous-oxy AHb1 showed an elution profile with a single peak (Figure 1A); the position of the peak changed with respect to protein concentration between that of a protein with an apparent molecular mass of ~20 kDa at low concentrations and that with a molecular mass of ~36 kDa at high concentrations. Since the theoretical calculated molecular mass of monomeric AHb1 is 18,034 Da and the crystal structure depicted a dimeric protein [21], it can be surmised that the observed elution profiles indicate a rapid exchange in the equilibrium between a monomeric and dimeric form of the protein (Figure 1A,B). Importantly, no other species were observed. The ratio of dimer to monomer increased as the protein concentration increased. The plot of the percent dimeric AHb1 as a function of the total AHb1 concentration (in dimer equivalents) yielded a hyperbolic curve and linearization of the curve gives a *K_d_* value of 1.2 ± 0.3 μΜ (Figure 1C).

SEC was also used to measure the *K_d_* value of AHb1 in its ferric form to explore the effect of O_2_ on the monomer-dimer equilibrium. Similar to ferrous-oxy AHb1, ferric AHb1 also displayed an equilibrium between a monomeric and dimeric species, at low and high protein concentrations, respectively; however, the *K_d_* obtained was 11 ± 1 μΜ (Figure 1D). Therefore, oxygen binding causes stabilization of the AHb1 dimer interaction, as reflected in an overall ~9-fold decrease in *K_d_*.

**Figure 2:**
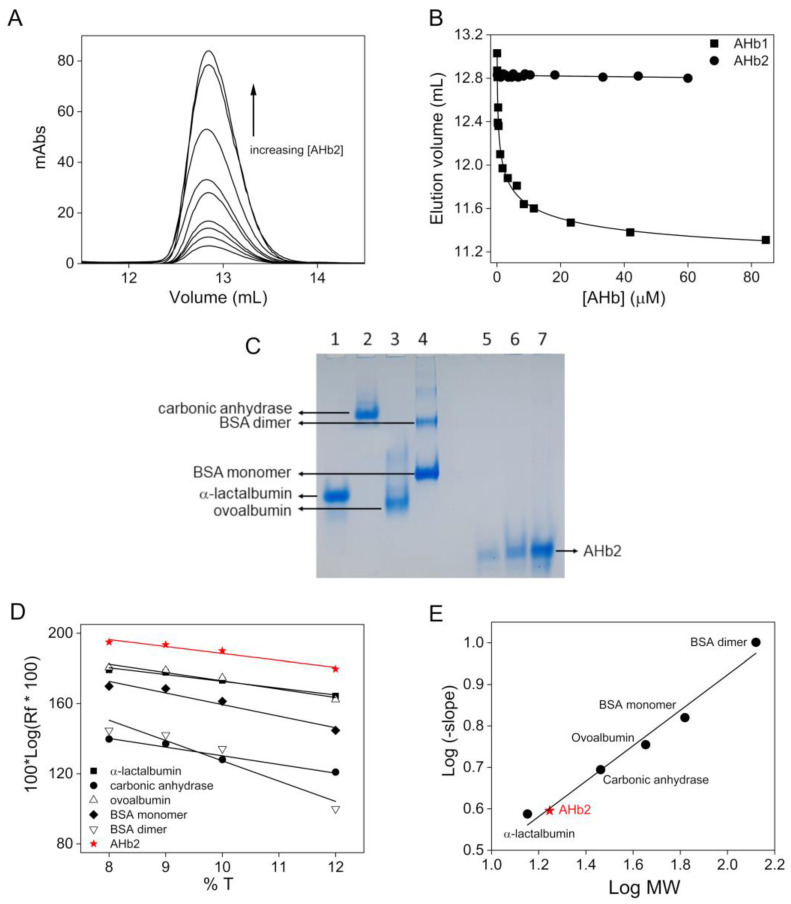
Oligomeric state of AHb2. (**A**) Representative elution profiles of ferric AHb2 on a Superdex-75 Increase 10/300 GL column at selected protein concentrations (0.1–60 μM range). The arrow indicates the direction of shift of the chromatographic peak with increasing protein concentration. (**B**) Comparison of AHb1 and AHb2 elution volumes at different protein concentrations. (**C**–**E**) Relative electrophoretic mobility calculation of AHb2 monomer by Ferguson plot. (**C**) Representative native-PAGE (9% T) showing the mobility of standard proteins and AHb2. Lane 1: α-lactalbumin; Lane 2: carbonic anhydrase; Lane 3: ovoalbumin; Lane 4: BSA (dimer and monomer); Lane 5: AHb2 5 μM; Lane 6: AHb2 10 μM; Lane 7: AHb2 25 μM. (**D**) Effect of %T on the relative mobility of standard proteins and AHb2. Plot according to Ferguson et al. [30] of 100 [log (Rf·100)] versus %T. (**E**) Standard curve obtained by plotting the log of the negative slope (from (**D**)) against the log of standard proteins molecular weight (MW). The graph was used to determine the apparent MW of AHb2 (red five-pointed star).

Parallel experiments were carried out with AHb2 which, due to the low oxygen equilibrium constant, was always present in the ferric form. The chromatographic profiles indicated the presence of a single peak at an elution volume corresponding to an apparent molecular mass of ~23 kDa, whose position did not change over the protein concentration range examined (0.1–60 μM) (Figure 2A). This behavior suggests that the observed peak represents a single species and not the equilibrium between two oligomeric forms as for AHb1 (Figure 2B). The expected molecular mass of AHb2 with one heme molecule is 17,871 Da. The results obtained are therefore consistent with a monomer in solution; no dimers or larger oligomers are present.

An additional estimate of the size of AHb2 in native conditions was obtained by analyzing the electrophoretic mobility (R_f_) of AHb2 and comparing it with that of standard proteins with known molecular masses at various polyacrylamide concentrations (8, 9, 10 and 12%) in native-PAGE (Figure 2C–E). Only one band was observed for AHb2 at either low (5 μM) or high protein concentrations (25 μM) which corresponds approximately to 18 kDa, a value that is fully consistent with a monomeric species. Thus, it can be concluded that AHb1 exists in solution as a monomer-dimer equilibrium, while AHb2 is present only as a monomer.

### 3.2. Mutational Analysis in the Dimerization Interface of AHb1

**Figure 3:**
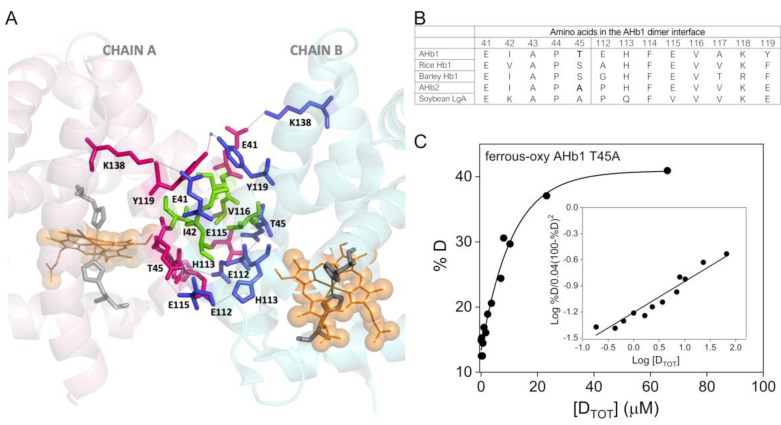
Dimeric interface of AHb1. (**A**) View of the dimeric protein showing the interface region in AHb1 (PDB 3ZHW). Chain A is in light pink and chain B is in light blue. Key residues involved in hydrogen bonding (dotted lines) and Y119-Y119 interaction (by water molecules) are labelled and represented as pink sticks (chain A) or blue sticks (chain B). Residues I42 and V116 in each monomer forming the hydrophobic cluster are in green. The heme molecules are represented as lines and spheres colored in orange. Distal and proximal histidine residues configuring the heme cavity are in gray sticks. (**B**) The AHb1 dimer interface regions aligned with homologous regions from other plant hemoglobins. (**C**) Representative dimer-monomer dissociation curve of ferrous-oxy AHb1 T45A at pH 7.2 in 150 mM Tris-acetate buffer pH 7.5. [D_TOT_] represents the total AHb1 T45A concentration (in dimer equivalents) and %D represents the percentage of protein that is actually dimer at various AHb1 T45A concentrations. Inset shows the plot of log %D/0.04 (100 − %D)^2^ versus log [D_TOT_] according to Manning et al. [28]. Procedures used for evaluation of *K_d_* values are described in detail in Section 2.5.

Crystallographic data of the dimeric form of AHb1 suggested a key role of the protein–protein interaction surface for AHb1 oligomerization (Figure 3A) [21]. More specifically, it was shown that the electrostatic interactions between the pairs T45–E115 and E112–H113 of each monomer in AHb1 and the water-mediated interaction with Y119 play crucial roles in promoting subunits association. Hydrophobic interactions involving I42 and V116 provide further stability to the dimeric interface [21].

Sequence alignment of the dimer interface region of plant Hbs showed that the residues that form this region in AHb1 are also present in other class 1nsHbs, such as those from rice, barley, and corn. This suggests that the dimeric interface might be important in influencing the role and chemical behavior of this class of proteins (Figure 3B). According to this hypothesis, AHb2 is predicted to be incapable of dimerization since it contains Ala and Pro at positions 45 and 112, respectively (numbering according to AHb1). Notably, soybean leghemoglobin, which is monomeric, also has an Ala residue at position 45, which likely prevents dimer formation (Figure 3B).

To test this hypothesis and the relevance of selected amino acids in the dimeric region, we mutated the critical Thr45 residue of AHb1 to Ala. To exclude that the mutation could impact the folding and structural integrity of the protein, absorbance spectra (Figure S1) and far-UV CD spectra (Figure S2A) for the mutant were recorded. The mutant showed no significant differences compared to the wild-type protein in either absorbance spectroscopic features or secondary structure elements (α-helical folds). Moreover, thermal stability studies by CD at 222 nm resulted in similar thermal denaturation profiles (Figure S2B), indicating that the mutation did not impact the stability of the protein. However, when we investigated the quaternary structure of AHb1 T45A in solution, we found that the T45A mutation resulted in a species that exists in a monomeric form at higher protein concentrations compared to wild-type protein. The *K_d_* measured from the elution profiles of ferrous-oxy AHb1 T45A was 876 ± 90 µM, which is ~730-fold higher than that of ferrous-oxy wild-type AHb1 (Figure 3C). Thus, Thr45 significantly contributes to AHb1 dimerization as its replacement with Ala produces a mostly monomeric protein that impairs a crucial interaction in the dimeric interface of AHb1.

In parallel, with the idea to evaluate the above-mentioned dimer interface fingerprint sequence to find residues that favor the dimer and disfavor the monomer, we generated a single mutant of AHb2 isoform by substituting its Ala at position 45 (numbering according to AHb1) to Thr, as found in AHb1, in order to recreate the electrostatic pair T45-E115. However, the mutation had no effect on the quaternary structure of AHb2 with the mutant protein, which remained in a monomeric form, as with wild-type AHb2, over all protein concentrations examined (data not shown). In addition to Ala, other differences in the dimer interface fingerprints are thus present in AHb2 that may prevent AHb2 dimerization.

### 3.3. Effects of Quaternary Structure on the Hexacoordination Process in AHb1

**Figure 4:**
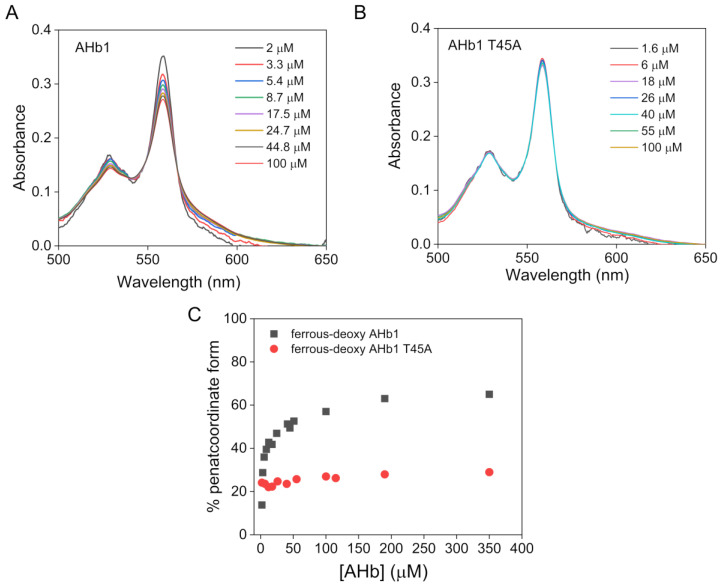
Effects of quaternary structure on the hexacoordination process in AHb1. Visible absorbance spectra for ferrous-deoxy wild-type AHb1 (**A**) and T45A mutant (**B**) proteins at selected representative protein concentrations (from 0.7 to 350 μM recorded in 0.1 M phosphate buffer, pH 7.0). (**C**) Plot of the % pentacoordinate form of AHb1 wild-type and AHb1 T45A estimated from the spectra in (**A**,**B**) versus protein concentration.

We next investigated the effect of protein concentration, and therefore oligomeric state, on the hexacoordination equilibrium in wild-type and T45A AHb1 proteins. It is well-known that AHb1 has a mixture of penta- and hexacoordinate heme and that the pentacoordinate form increases with increasing protein concentration [16,17,23]. We estimated the fraction of pentacoordinate species at different concentrations of ferrous-deoxy wild-type AHb1 and T45A mutant taking the ratio of absorbance at 555 nm to that at 540 nm as an indicator of the degree of heme hexacoordination [32] and using deoxy-AHb2 reference spectrum for a pure hexacoordinate species and the AHb1 E7L spectrum (mutation of the distal histidine HisE7 to Leu) for the fully pentacoordinate form as described elsewhere [16]. Figure 4A shows several ferrous-deoxy AHb1 spectra (for clarity) at different protein concentrations. We confirmed that the pentacoordinate form of AHb1 increased with increasing protein concentration (from ~13% at 1 μM to ~65% at 190 μM) (Figure 4C), suggesting that the penta- and hexacoordinate form in AHb1 is coupled to its oligomeric state. Of note, the same analysis performed on the AHb1 T45A mutant showed that the fraction of pentacoordinate species is lower in the mutant compared to wild-type AHb1 and, most importantly, does not significantly increase with increasing protein concentration in the range tested (pentacoordinate form is ~26% from 0.7 to 350 μM) (Figure 4B,C).

### 3.4. CD spectra of AHb1 and AHb2

The measurement of CD spectra to explore the structural organization of hemoglobins is of particular advantage due to the presence of the heme group, which is chiral when free in solution, but gives rise to a dichroic signal when located in an asymmetric environment within the globin moiety. Therefore, the signals typical of the different regions, arising from plane polarized π-π^*^ transitions, i.e., L-band (near-UV, 240–320 nm), Soret or B-band (300–450 nm), and Q-bands (visible region, 450–650 nm) allow the description of some structural determinants of the AHb1 and AHb2 isoforms that are impossible to dissect using only electronic absorption spectroscopy.

**Figure 5:**
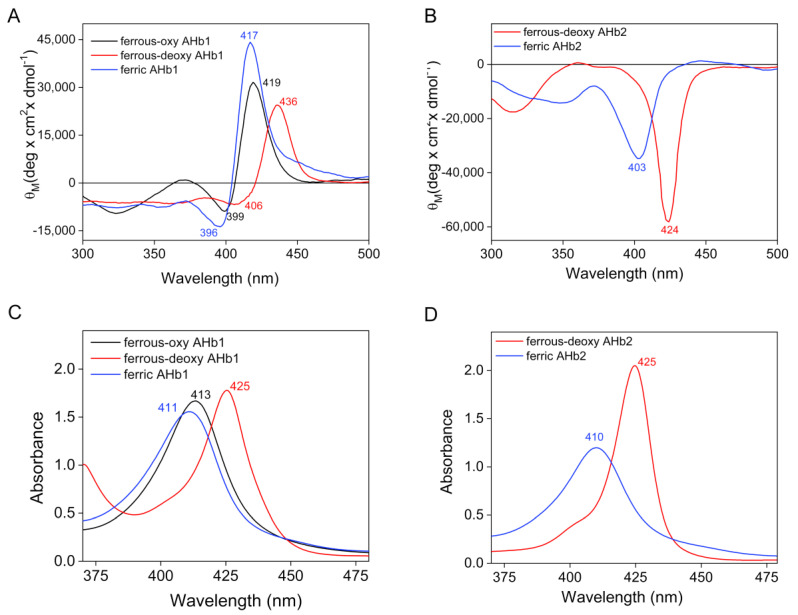
Circular dichroism (**A**,**B**) and absorption (**C**,**D**) spectra of AHb1 and AHb2 in the Soret region. CD spectra (**A**,**B**) were recorded using 33 µM heme proteins in 10 mM Tris-HCl buffer, pH 8.0, and absorption spectra (**C**,**D**) using 20 µM heme proteins in 20 mM Tris-HCl buffer, pH 8.0.

Figure 5 shows the CD Soret-band (Figure 5A,B) and absorption spectra (Figure 5C,D) of AHb1 and AHb2, respectively. Modifications in the CD Soret region are mainly ascribed to the coupling of the heme π-π* transitions with the π-π* transitions of neighboring aromatic amino acids and to an altered spatial orientation of these residues in relation to heme [33]. However, the contributions of polarizable groups and the heme distortions from planarity have also been invoked [34]. The CD spectra of AHb1 and AHb2 reveal pronounced differences in the 300–450 nm region, supporting the notion that the sensitivity of the Soret CD bands to differences in heme environments can be of great value in assessing differences and similarities when comparing various hemoglobins.

The CD spectra of AHb1 displayed an asymmetric couplet (two oppositely signed CD bands split in energy corresponding to two transition dipole moments, B_x_, B_y_ in the porphyrin plane) in the Soret region in all the oxidation and ligand states examined (i.e., ferrous-oxy, ferrous-deoxy and ferric) (Figure 5A). The major positive Soret CD band varies in position with the oxidation states of the protein from the 419 nm-band for the ferrous-oxy form to 436 nm for the ferrous-deoxy to 417 nm-band for the ferric species. The negative component, which is less intense, is centered at 399, 406 and 396 nm, for the ferrous-oxy, ferrous-deoxy, and ferric species, respectively. In all cases, the positive CD band of the couplet was 8–11 nm red-shifted with respect to the corresponding position of the absorption maxima, which were located at 413, 425, and 411 nm for the ferrous-oxy, ferrous-deoxy, and ferric species of the protein, respectively (Figure 5C).

In contrast to AHb1, the Soret CD spectra for AHb2 (Figure 5B) displayed a major negative extremum that does not seem to be split into the two energetic components. The negative maxima are found at 424 nm for the reduced ferrous-deoxy species and 403 nm for the oxidized ferric species. The corresponding positions of the absorption maxima are located at 425 and 410 nm for the ferrous-deoxy and ferric species of the protein, respectively (Figure 5D).

**Figure 6:**
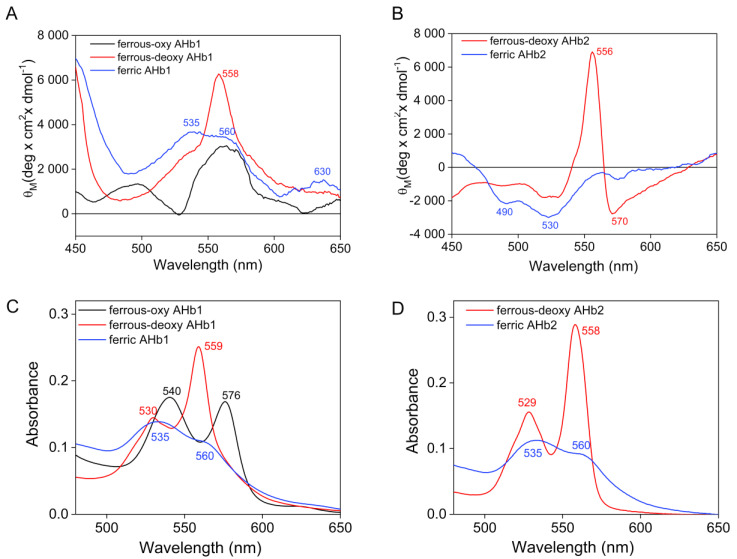
Circular dichroism (**A**,**B**) and absorption (**C**,**D**) spectra of AHb1 and AHb2 in the visible region. CD spectra (**A**,**B**) were recorded using 120 μM (AHb1) and 140 μM (AHb2) heme in 10 mM Tris-HCl buffer, pH 8.0. For absorbance measurements (**C**,**D**), protein concentrations were 20 µM heme proteins in 20 mM Tris-HCl buffer, pH 8.0.

We also examined the CD spectral properties of AHb1 and AHb2 in the visible region (450–650 nm) (Figure 6). In this region, only the heme-associated electronic transitions (Q bands, α and β) are predicted to contribute [33].

The visible CD spectra of AHb1 showed multiple positive bands whose intensity and maximum position vary with the redox state of the iron (Figure 6A). The reduced deoxy form showed a major peak and a shoulder centered at 558 and ~535 nm, respectively, which can be attributed to the α and β bands (or Q_0_ and Q_v_), respectively. The high value of the β component with respect to the α component, also corroborated by the absorbance electronic spectra (Figure 6C), can be interpreted as indicative of a highly asymmetric heme environment [35], and in particular to the asymmetry of the proximal bond [36,37]. The ferric species was characterized by two corresponding α and β dichroic signals at ~560 and ~535 nm, respectively, that exhibit a lower intensity, and an additive band at 630 nm that supports the presence of a fraction of high spin pentacoordinate ferric species.

Similar to the Soret region, the visible CD signals for AHb2 were substantially negative, and especially for the ferric derivative (Figure 6B). However, the deoxy-ferrous form possessed both negative and positive peaks, with a pronounced positive band at 556 nm that seems to result from a splitting of a signal with its negative component centered at ~570 nm. The well-defined dichroic spectral bands of AHb2 (with respect to those of AHb1) and the absence of the charge transfer band at low frequency (~630 nm) are in accordance with the presence of a pure hexacoordinate species.

**Figure 7:**
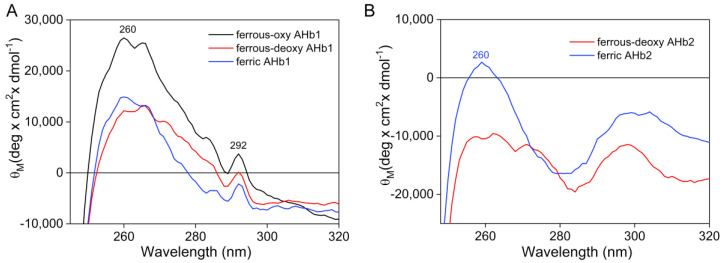
CD spectra of AHb1 (**A**) and AHb2 (**B**) in the near-UV region. Protein concentrations were 30 µM heme, in 10 mM Tris-HCl buffer, pH 8.0.

Major spectral differences between AHb1 and AHb2 were also present in the near-UV region (240–325 nm) (Figure 7). In this region, the heme bands are poorly characterized because they overlap in part with the UV absorption of the aromatic side chains of the protein moiety.

The near-UV CD spectra of ferrous-deoxy, ferrous-oxy, and ferric AHb1 (Figure 7A) showed a prominent positive L-band (with a maximum around 260 nm) whose intensity is affected by the ligand and the redox state, being less intense in the ferrous and ferric forms and more pronounced when O_2_ is bound. As the band decreases in the apoprotein (Figure S3), it can be ascribed to the optical activity of the heme group. In addition, a peak at 292 nm was present in all AHb1 forms, comprising the apoprotein (Figure S3), and can be attributed to a heme-hydrogen bonded tryptophan residue [38] that is rigidly held in a non-polar environment. Interestingly, upon deoxygenation, the positive CD signal at 292 nm became negative, likely suggesting that changes in the protein’s tertiary structure following oxygen binding can alter the local environment surrounding the Trp residues.

The near-UV CD spectra of AHb2 (Figure 7B) were completely different from those of AHb1. In comparison to AHb1, the band at 260 nm was weaker in all AHb2-derivative spectra and it was difficult to individuate a definite band as there were multiple negative maxima. Interestingly, the signal at 292 nm was not present.

## 4. Discussion

The understanding of the biological role of the nsHbs has been a relevant topic for several years. In this scenario, comparison of structural parameters of different nsHbs within one plant species may help to confirm the specific functions of nsHbs hypothesized in plants.

Herein, we expand upon previous biophysical studies on AHb1 and AHb2 by identifying specific structural features of these two isoforms using SEC and CD spectroscopy. From the results presented herein, it appears that the differences in globin structure and the environment surrounding the heme groups in AHb1 and AHb2 manifest not only in different physical properties, such as oxygen affinity and the degree of hexacoordination, but are also revealed in their CD properties and different oligomeric state.

CD spectroscopy is a valuable method to analyze the optical activity of heme proteins deriving from diverse types of heme-protein interactions. Major spectral differences between AHb1 and AHb2 appear mainly in the Soret region around 400 nm and in the near-UV around 260 nm. In particular, a striking difference between the Soret CD spectra of AHb1 and AHb2 is their opposite sense. Indeed, the CD spectra of AHb1 displayed an asymmetric couplet with a major positive band, while the Soret CD spectra for AHb2 displayed a major negative extremum that does not seem to be split into the two energetic components. While the splitting of the Soret band is visible in the CD spectra of cytochrome c [39], it is not present in myoglobin, where a shift of CD spectra is evident in the ferrous form (red shift) and cyanomyoglobin (blue shift) [40]. For all the myoglobin derivatives, the observed Cotton effect was dominated by one component of the Soret state (B_y_) [40]. In the case of AHb1, the observed noncoincidence of the absorption and CD maxima and the presence of a couplet unambiguously reveal a splitting of the B state associated with the interactions between heme and the protein matrix. Of note, this splitting is diagnostic of reduced degeneration of the energetic components contributing to the Soret signal, which can be translated into a less symmetric heme microenvironment, thus suggesting the presence of a strong network of interactions between the heme moiety and the protein. This conclusion is further supported by comparison of CD spectra of AHb1 wild-type with those of the fully pentacoordinate AHb1 E7L mutant in their ferric forms (Figure S4). Indeed, the mutant displays a blue-shifted Soret band splitting centered at ~390 nm (positive and negative maxima at 400 and 372 nm, respectively) with the two components of the couplet that are comparable in magnitude. These differences are ascribable to the presence of distinct ligands in the distal cavity (substitution of the distal histidine with a leucine) which can affect the two transition dipole moments, B_x_ and B_y_, in the porphyrin plane and, thus, alter heme-protein interactions. Moreover, a 340 nm band, which is typical of high spin pentacoordinate species and is also present in myoglobin [41], becomes evident when the distal His is absent, confirming not only the pentacoordinate nature of this mutant but also the existence of a different electronic configuration in the mutant and wild-type proteins.

The lack of complete coincidence between absorbance and CD maxima, in particular for the ferric form, support a splitting of the excited B-band also for AHb2, even if the couplet is absent. Indeed, the algebraic sum of the components of different energy levels could mask the second positive component. However, the CD Soret band of deoxy-ferrous AHb2 is only one nm blue-shifted compared to the absorbance spectrum, likely indicating a more symmetric heme environment and a weaker interaction between the heme moiety and the protein than in AHb1.

Of note, while vertebrate hemoglobins and myoglobins exhibit large positive ellipticities in the Soret region, negative Cotton bands have been observed in symbiotic hemoglobins [37,38,42,43]. In particular, the Soret CD spectra of AHb2 show striking similarities in both shape and size to those of other species of leghemoglobins, being not only qualitatively but also quantitatively very similar to those of leghemoglobin from soybean [37,42,43], and thus indicating that the two proteins have a similar heme environment. These conclusions are further supported by analysis of the CD spectra in the visible region. The comparison of the ferrous and ferric CD spectra of AHb1 with those of vertebrate hemoglobins and myoglobins [36,44,45,46] reveals a clear similarity between the visible CD region of these proteins, while the negative visible CD patterns of AHb2, and in particular their shapes and minims (particularly for the ferric derivative) are reminiscent of those of leghemoglobins [38,47].

The existence of differences in the heme environment between AHb1 and AHb2 is also evident by CD signals in the near UV region, with AHb1 spectra showing a positive CD band around 260 nm and AHb2 showing a smaller negative CD envelope. The positive CD band at 260 nm is generally ascribed to the heme moiety (as supported by its decrease in the AHb1 apoprotein, Figure S3) and is influenced by the spin and coordination state of the iron. A similar positive CD band was reported for myoglobin and for the heme cytochrome c undecapeptide, which does not possess aromatic residues [38,48]. On the other hand, AHb2 displayed a behavior that was more similar to that observed for leghemoglobins [38], with a smaller heme contribution to the 260 nm band, suggesting that the heme group is in closer contact and more strongly bound to the protein matrix in AHb1 than in AHb2. Interestingly, a positive band at 292 nm is visible in all AHb1 derivatives (comprising the apoprotein), while it is absent in AHb2. This band is also present in soybean leghemoglobin [38] and is attributed to a tryptophan residue. By comparing the primary sequences of AHb1, AHb2, and soybean leghemoglobin there is strong evidence that Trp132 will be primarily responsible for this CD signal in AHb1 (Trp121 in soybean leghemoglobin) which is replaced by Tyr129 in AHb2 (Figure S5), being in a non-polar environment similar to that in leghemoglobin.

Overall, our comparative CD analysis indicates that the major spectral differences between AHb1 and AHb2 originate from the mode of interaction between the heme and the surrounding protein side chains and suggest that the two isoforms are more related to hemeproteins belonging to different evolutionary groups: AHb1 shares some properties with myoglobin and hemoglobins, while AHb2 presents a significant structural correspondence to leghemoglobins in the pattern of interactions between the heme and the microenvironment. These findings confirm the leghemoglobin-like functional and structural properties of AHb2 and argue in favor of the proposal that the symbiotic hemoglobins of legumes arose from a class 2 non-symbiotic gene [4,6,13,14].

In addition to differences in the heme microenvironment, we also found striking differences in the oligomeric state of AHb1 and AHb2. Plant Hbs have different quaternary structure, comprising monomeric leghemoglobins [3], tight dimers (*K_d_*, dimer < 1 µM) as in *Parasponia andersonii* Hb and *Trema tomentosa* Hb [9], and more weak dimers (*K_d_*, dimer ~80 µM) as in rice Hb1 and other nsHbs [49,50]. Our analysis of the quaternary structure of AHb1 and AHb2 showed that AHb1 in solution exists as a monomer-dimer equilibrium, with the dimeric species dominating at high protein concentrations, while AHb2 is present only as a monomer. Interestingly, the finding that AHb2 is monomeric as are leghemoglobins is highly consistent with our CD analysis, corroborating the structural similarity between class 2 nsHbs and leghemoglobins.

The *K_d_* value obtained for dimerization for ferrous-oxy AHb1 was ~1 μM, indicating that below this concentration the equilibrium shifts towards the monomeric form, while above 1 μM the dimer is the predominant equilibrium species. Such an equilibrium could allow for an efficient mechanism to sense protein concentrations at physiological pH. Dimerization is a common phenomenon for many globins with an impact on their biological role and regulation. Indeed, only some Hbs exhibit a quaternary structure, and thus there is most likely a reason for that. Possible explanations include cooperative ligand binding and stability. However, cooperative ligand binding has never been observed in nsHbs. Moreover, a possible contribution of quaternary structure to protein stability seems improbable since proteins with different quaternary states (e.g., nsHbs and leghemoglobins) are naturally stable and mutant proteins that exist as monomeric species (e.g., AHb1 T45A, and others [49]) show no significant differences compared to the wild-type protein in their stability profiles.

Previous studies on rice Hb1, which exists in solution as a monomer-dimer equilibrium as AHb1, have shown that ligand binding kinetics are not substantially influenced by quaternary structural modifications [49], thus excluding the possibility that the oligomeric state in nsHbs class 1 could be related to a potential oxygen transport function for these proteins. Our results suggest that quaternary structure could play a role in controlling the hexacoordination process in AHb1. Indeed, the pentacoordinate form is stabilized in the dimer compared to the monomer, suggesting that the concentration dependent formation of the penta- and hexacoordinate form in AHb1 is linked to the formation of the dimer and the monomer, respectively. It should be noted that the dimer interface in nsHbs class 1 is highly conserved, suggesting that it likely has some role in the chemical behavior and physiological functions of this class of proteins. Based on our results, it is possible that the dimeric interface has a role in modulating the hexacoordination equilibrium. Accordingly, the replacement of the residue Thr45 with Ala within the dimer interface produced a mostly monomeric protein with an equilibrium shifted toward the hexacoordinate form compared to wild-type AHb1. These findings are fully consistent with data obtained with rice hemoglobin Hb1 [51] and the hypothesis about the crucial role of the CD region, which is mainly involved in the dimeric interface, in defining the coordination state of globins [52,53].

The *K_d_* observed for dimerization of AHb1 is consistent with a mostly dimeric protein in our experiments. However, in plants, the local concentration of AHb1 is probably lower. Thus, care should be taken in assessing the relevance of our in vitro results of diverse oligomeric states to in vivo conditions. Nonetheless, our results could suggest that in vivo the oligomerization properties of AHb1 could fulfill the need to maintain a fraction of pentacoordinate protein both at resting and under stress conditions, thus allowing the protein to adopt a more reactive state.

## 5. Conclusions

Overall, our results point to a more comprehensive understanding of the structure–function and evolutionary relationships of AHb1 and AHb2 and more generally of nsHbs in plants. However, there is still ample work to be done to obtain a clear-cut picture of the structures and ligand binding properties and for complete understanding of the physiological functions of these plant proteins.

## Supplementary Materials

The following are available online at https://www.mdpi.com/2218-273X/10/12/1615/s1, Figure S1: Absorbance spectra of AHb1 T45A, Figure S2: Far-UV CD spectra and thermal denaturation of AHb1 and AHb1 T45A, Figure S3: CD spectra of ferrous-oxy AHb1 and apo-AHb1 in the near-UV region, Figure S4: CD spectra of ferric AHb1 wild-type and ferric AHb1 E7L mutant in the 250–500 nm region, Figure S5: Multiple sequence alignments of AHb1, AHb2 and leghemoglobin.
