# Supplementary material for: Structural Insights into the Heme Pocket and Oligomeric State of Non-Symbiotic Hemoglobins from Arabidopsis thaliana
Source: Biomolecules. 2020 Nov 29;10(12):1615. doi: 10.3390/biom10121615 (PMC7761212; doi:10.3390/biom10121615)
Supplement: Supplementary file 1 [file biomolecules-10-01615-s001.pdf]

Supplementary Material

Structural insights into the heme pocket and oligomeric state of non-symbiotic hemoglobins from *Arabidopsis thaliana*

Alessandra Astegno[1], Carolina Conter[1], Mariarita Bertoldi[2], Paola Dominici[1*]

[1] Department of Biotechnology, University of Verona, Strada Le Grazie 15, 37134, Verona, Italy.
[2] Department of Neuroscience, Biomedicine and Movement Sciences, Section of Biological Chemistry, University of Verona, Strada Le Grazie, 8, 37134, Verona, Italy.

*Corresponding author: paola.dominici@univr.it

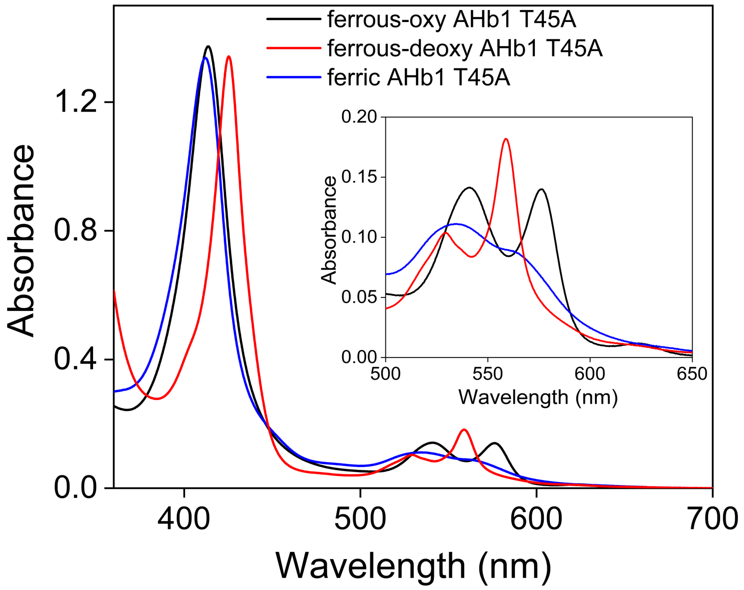

Figure S1. Absorbance spectra of AHb1 T45A. Absorption spectra of 20 µM ferrous-oxy (black line), ferrous-deoxy (red line) and ferric (blue line) AHb1 T45A.

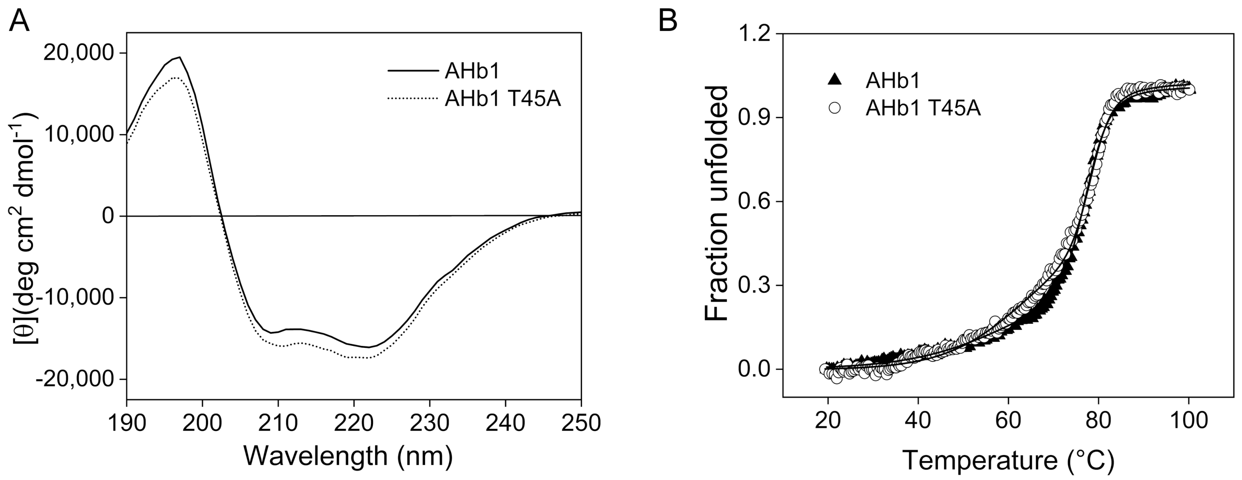

Figure S2. Far-UV CD spectra and thermal denaturation of AHb1 and AHb1 T45A. (**A**) Far-UV CD spectra of AHb1 (solid line) and AHb1 T45A (dotted line) recorded in 10 mM Tris-HCl pH 8. (**B**) Representative thermal denaturation profiles of AHb1 (solid triangles) and AHb1 T45A (open circles) recorded following ellipticity signal at 222 nm at 0.2 mg/mL protein concentration in 10 mM Tris-HCl pH 8.

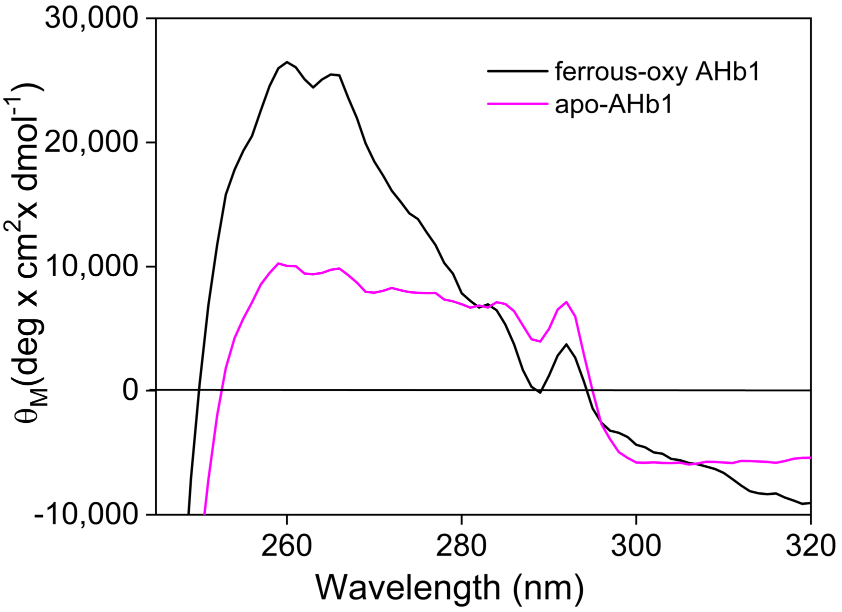

Figure S3. **CD spectra of ferrous-oxy AHb1 and apo-AHb1 in the near-UV region.**

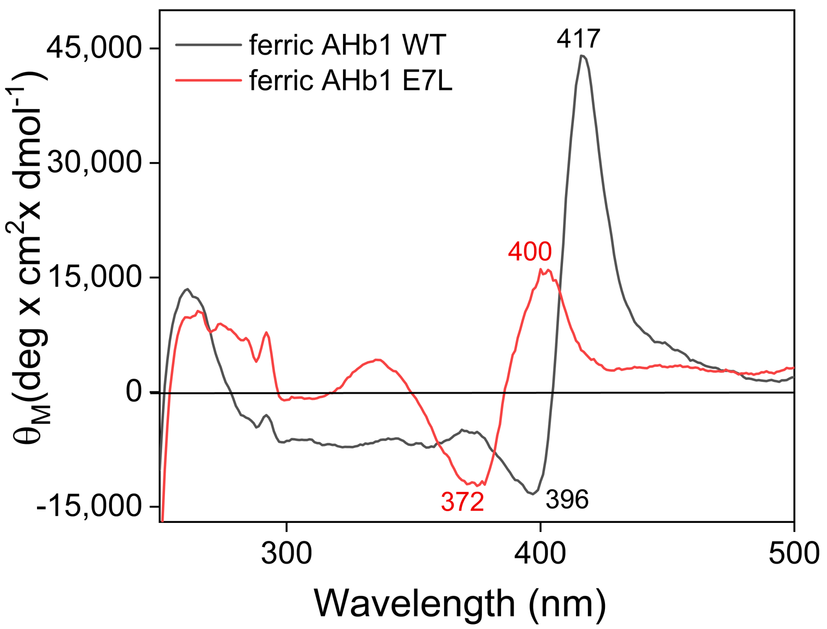

Figure S4. **CD spectra of ferric AHb1 wild-type and ferric AHb1 E7L mutant in the 250-500 nm region.**

```
LBA         -----MVAFTEKQDALVSSSFEAFKANIPQYSVVFYTSILEKAPAAKDLFSFLANGVD--        53
AHb1        MESEGKIVFTEEQEALVVKSWSVMKKNSAELGLKLFIKIFEIAPTTKKMFSFLRDSPIPA        60
AHb2        ---MGEIGFTEKQEALVKESWEILKQDIPKYSLHFFSQILEIAPAAKGLFSFLRDSDEVP        57
                  : ***:*:*** .*:. :* :  : .: :: .*:* **::* :**** :.

LBA         PTNPKLTGHAEKLFALVRDSAGQLKASGTVVAD----AALGSVHAQKAVTDPQFVVVKEA        109
AHb1        EQNPKLKPHAMSVFVMCCESAVQLRKTGKVTVRETTLKRLGASHSKYGVVDEHFEVAKYA        120
AHb2        HNNPKLKAHAVKVFKMTCETAIQLREEGKVVVADTTLQYLGSIHLKSGVIDPHFEVVKEA        117
              ****. ** .:* :  ::* **:  *.*..       **: * : .* * :* *.* *

LBA         LLKTIKAAVGDKWSDELSRAWEVAYDELAAAIKKA------   144
AHb1        LLETIKEAVPEMWSPEMKVAWGQAYDHLVAAIKAEMNLSN-   160
AHb2        LLRTLKEGLGEKYNEEVEGAWSQAYDHLALAIKTEMKQEES   158
            **.*:* .: : :. *:. **  ***.*. ***
```

Figure S5. Multiple sequence alignments of AHb1, AHb2 and leghemoglobin. AHb1 (*Arabidopsis thaliana* non-symbiotic hemoglobin class 1, UniProt: O24520), AHb2 (*Arabidopsis thaliana* non-symbiotic hemoglobin class 2, UniProt: O24521) and LBA (*Glycine max* leghemoglobin, UniProt: P02238).
